# Ticks and Texas Fever: Dr. Rogers’ Paper

**Published:** 1892-04

**Authors:** 


### THE TICK THEORY.

Dr. Rogers' reply to our burlesque report in last issue, will be read with interest.

We disclaim here any thought of discourtesy to Mr. Rogers, and especially, as he is an Episcopal minister, a fact unknown to us at the time of writing.

The subject has assumed a scientific importance far beyond our conception; we did not know that it was seriously entertained,—it looks so absurd on the face of it, but supposed Mr. Rogers was a stockman, who, without scientific knowledge, had gotten this idea into his head and was making a hobby of it. No, the editor does not yet "see where the laugh comes in." Until Mr. Rogers, who says "the disease is not caused by bacteria" enlightens us on his method of diagnosing the Texas cattle fever "by an examination of the blood" his position cannot be taken as proved. If the disease be infectious, until the specific organism of infection is demonstrated, no "examination of the blood" will furnish evidence of the infectious nature of the disease.

Mr. Rogers says: "Its nature (the disease) cannot be understood by supposing a simple transfer of bacteria from Southern cattle to pastures and from pastures to Northern cattle;" but he attempts to explain it by "a simple transfer of *ticks* from Southern cattle to pasture and from pasture to Northern cattle." It seems the Doctor gets ticks and bacteria mixed up.

The subject is a very important one, and if there is anything in it,—and there is certainly a remarkable coincidence of the fever with the presence of ticks, it is "worthy of scientific investigation." We publish the Doctor's article with pleasure.

